# Supplementary material for: Genomic analysis of global Plasmodium vivax populations reveals insights into the evolution of drug resistance
Source: Nat Commun. 2024 Dec 30;15:10771. doi: 10.1038/s41467-024-54964-x (PMC11685768; doi:10.1038/s41467-024-54964-x)

SUPPLEMENTARY INFORMATION

Genomic analysis of global *Plasmodium vivax* populations reveals insights into the evolution of drug resistance

Gabrielle C. Ngwana-Joseph[1], Jody E. Phelan[1], Emilia Manko[1], Jamille G. Dombrowski[1,2], Simone da Silva Santos[3], Martha C. Suarez-Mutis[3], Gabriel Vélez-Tobón[4], Alberto Tobón Castaño[4], Ricardo Luiz Dantas Machado[5], Claudio R. F. Marinho[2], Debbie Nolder[6], François Nosten[7,8], Colin J. Sutherland[1,6], Susana Campino[1,*], Taane G. Clark[1,9*]

[1]Department of Infection Biology, Faculty of Infectious and Tropical Diseases, London School of Hygiene and Tropical Medicine, Keppel Street, London, WC1E 7HT, UK

[2]Department of Parasitology, Institute of Biomedical Sciences, University of São Paulo, São Paulo, Brazil

[3]Oswaldo Cruz Foundation – Fiocruz – Rio de Janeiro, Brazil.

[4]Grupo Malaria, Facultad de Medicina, Universidad de Antioquia, Antioquia, Colombia

[5]Centro de Investigação de Microrganismos – CIM, Departamento de Microbiologia e Parasitologia, Universidade Federal Fluminense, Brazil

[6]UK Health Security Agency, Malaria Reference Laboratory, London School of Hygiene and Tropical Medicine, Keppel Street, London, WC1E 7HT, UK

[7]Centre for Tropical Medicine and Global Health, Nuffield Department of Medicine, University of Oxford, Oxford, OX3 7BN, UK

[8]Shoklo Malaria Research Unit, Mahidol–Oxford Tropical Medicine Research Unit, Faculty of Tropical Medicine, Mahidol University, Mae Sot, 63110, Thailand

[9]Faculty of Epidemiology and Population Health, London School of Hygiene and Tropical Medicine, London, WC1E 7HT, UK

*Joint Corresponding Authors:

Correspondence to Taane G. Clark or Susana Campino

Taane.Clark@lshtm.ac.uk / Susana.Campino@lshtm.ac.uk

Department of Infection Biology, Faculty of Infectious and Tropical Diseases

London School of Hygiene and Tropical Medicine, Keppel Street, London, UK

Fig S1. Chloroquine resistance status study design

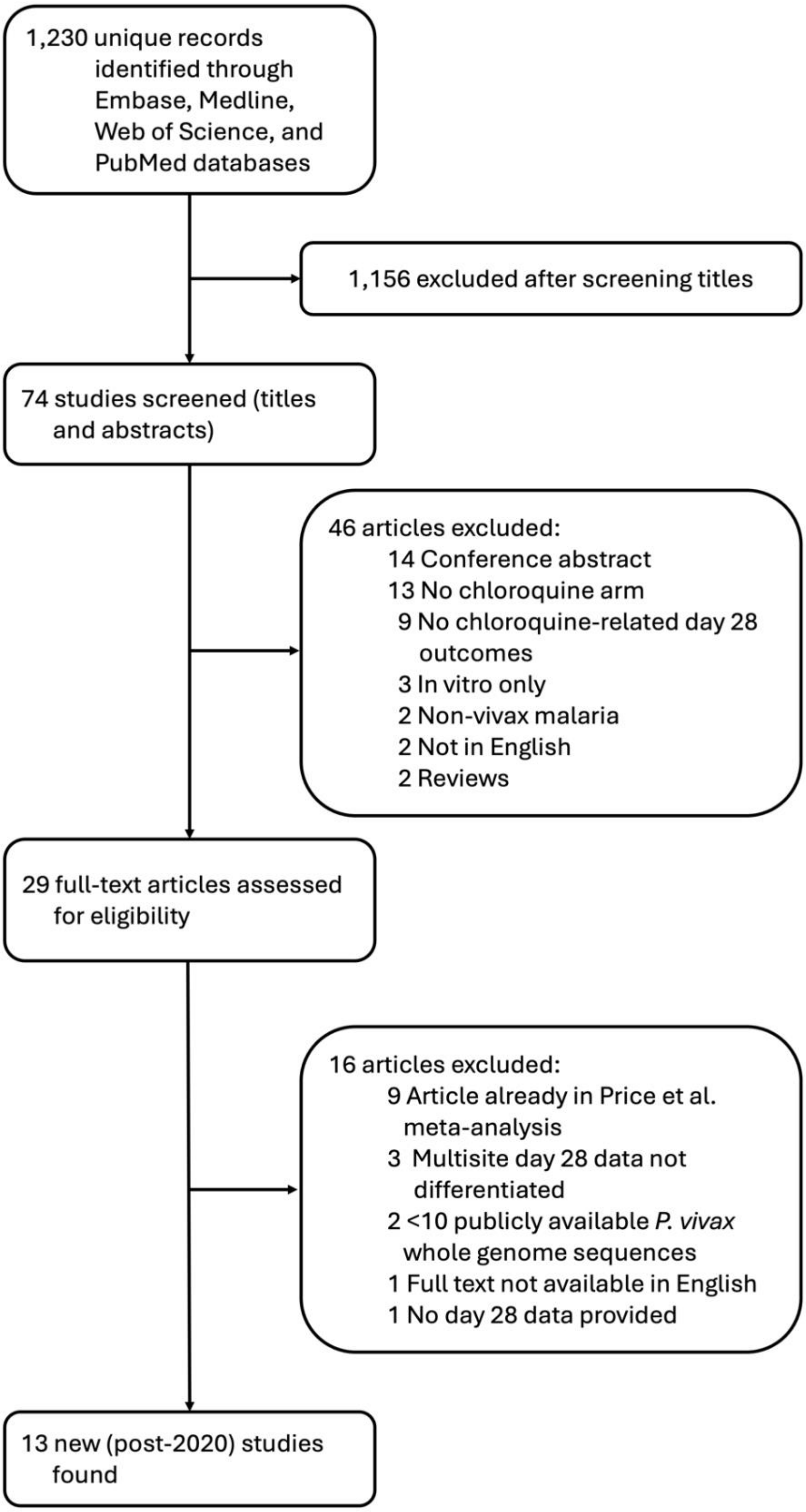

Figure S2. Within-sample diversity (F_{WS}).

Boxplots, coloured by subregional grouping, of the distribution of F_{WS} values in countries with more than 10 isolates. All boxplots contain boxes of the median and interquartile range (IQR), and whiskers extending to extreme datapoints less than 1.5 times the IQR. Outliers are represented as black dots.

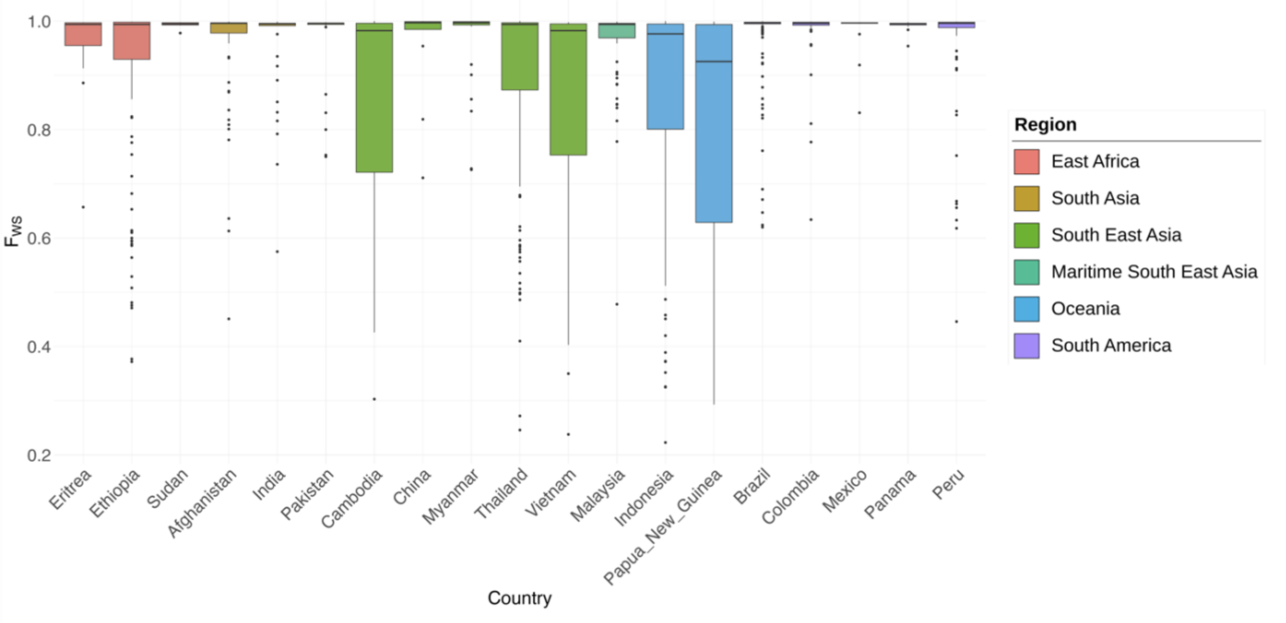

Figure S3. Ancestry analysis of *P. vivax* isolates (*N* = 1078) at the country level.

Stacked bar chart representing the proportion of each ancestral population (K1-K10) within each country with >10 isolates.

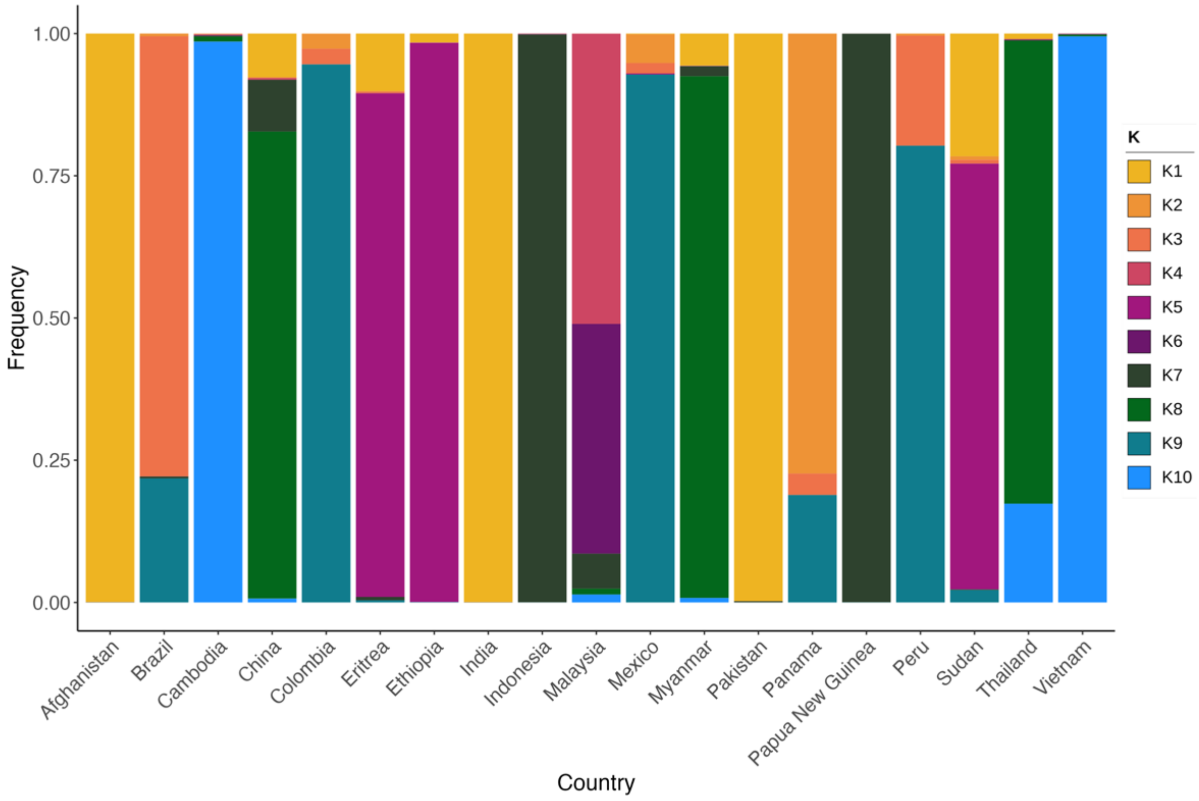

Figure S4. Principal component analysis (PCA) of 1,534 *P. vivax* isolates based on 499,206 high-quality bi-allelic SNPs generated using a pairwise SNP distance matrix.

Each isolate is coloured by the maximum *K* value, where maximum *K* corresponds to the predominant ancestral population.

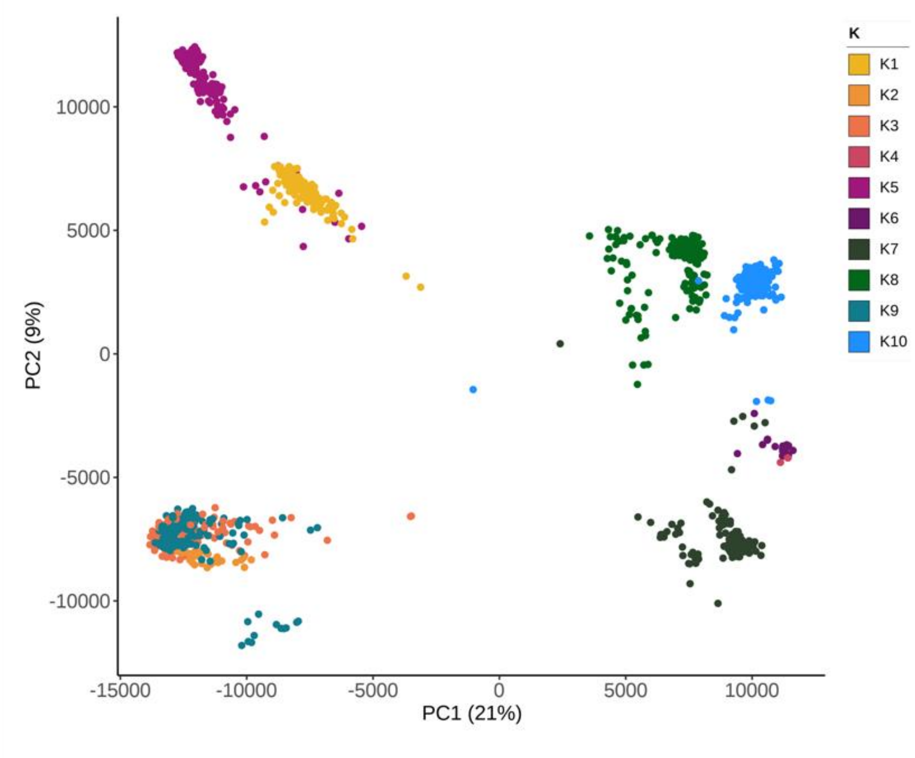

Figure S5. Relatedness between *P. vivax* isolates.

Distribution of pairwise identity-by-descent (IBD) fractions at a country level in countries with at least 10 isolates. All isolates except for Malaysian, Mexican, and Panamanian are represented in panel (**a**). The remaining panels represent pairwise IBD fractions in the highly related populations: (**b**) Malaysia, (**c**) Mexico, and (**d**) Panama.

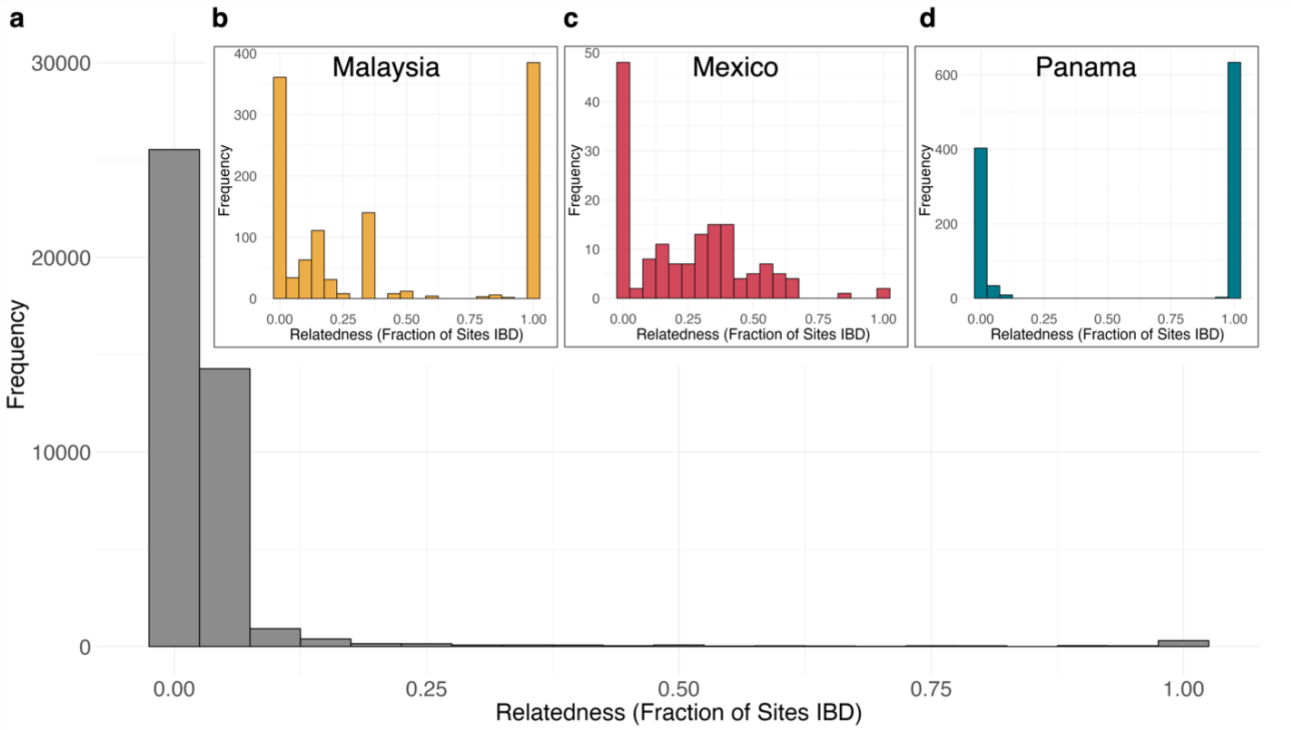

Figure S6. Genome-wide distribution of pairwise identity-by-descent (IBD) fractions calculated across sliding windows of 10 kb at a country-level in *P. vivax* isolates.

Chromosomal boundaries are denoted by vertical grey dashed lines.

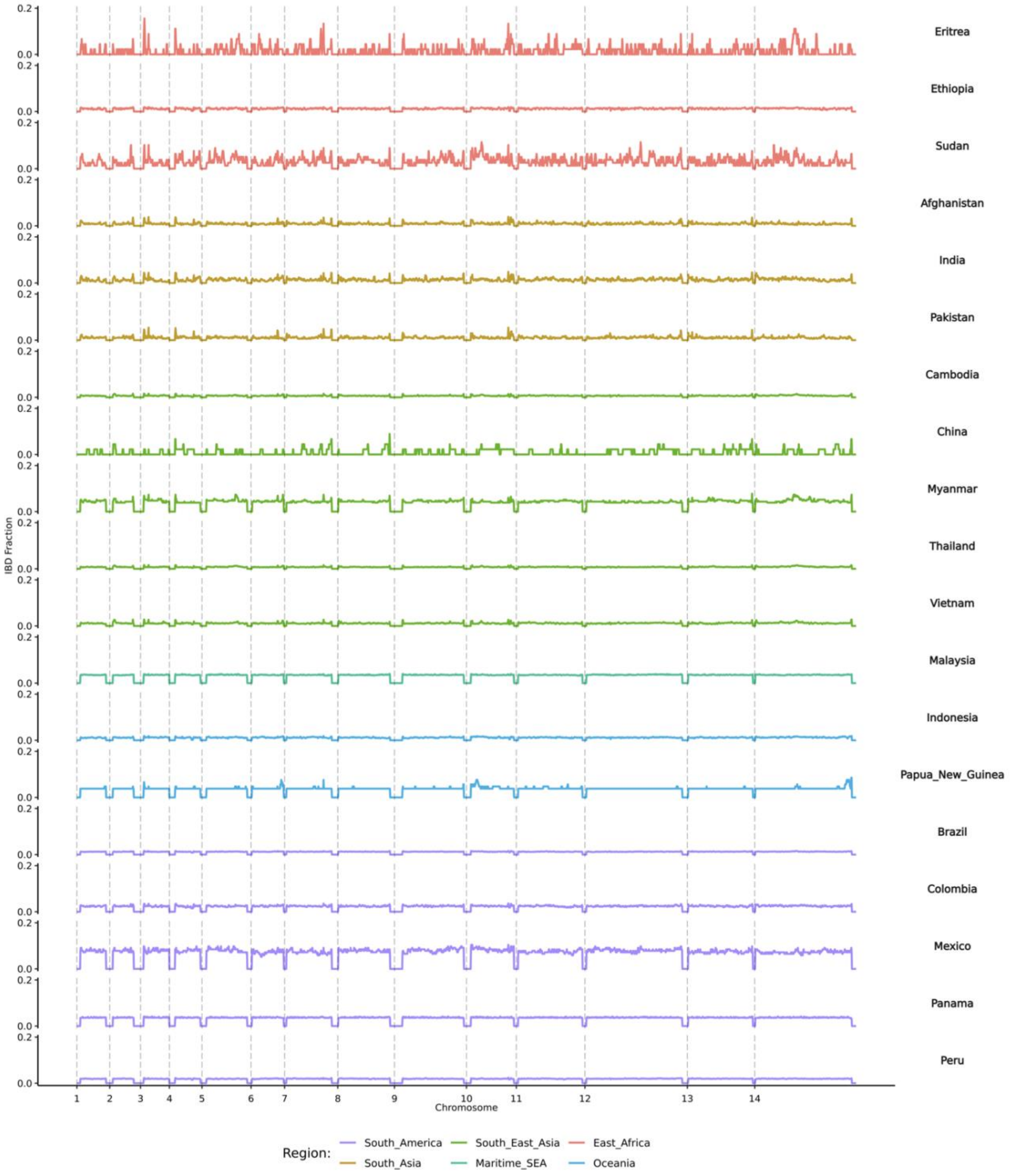

Figure S7. Genome-wide distribution of pairwise identity-by-descent (IBD) fractions at a country-level in *P. vivax* isolates.

Only populations with ≥10 monoclonal isolates are displayed. Boxes, coloured by subregional grouping, contain lines representing the median, lower quartile, and upper quartile. Whiskers extend to IBD values that are at a maximum of 1.5x the interquartile range. Outliers are represented as single points.

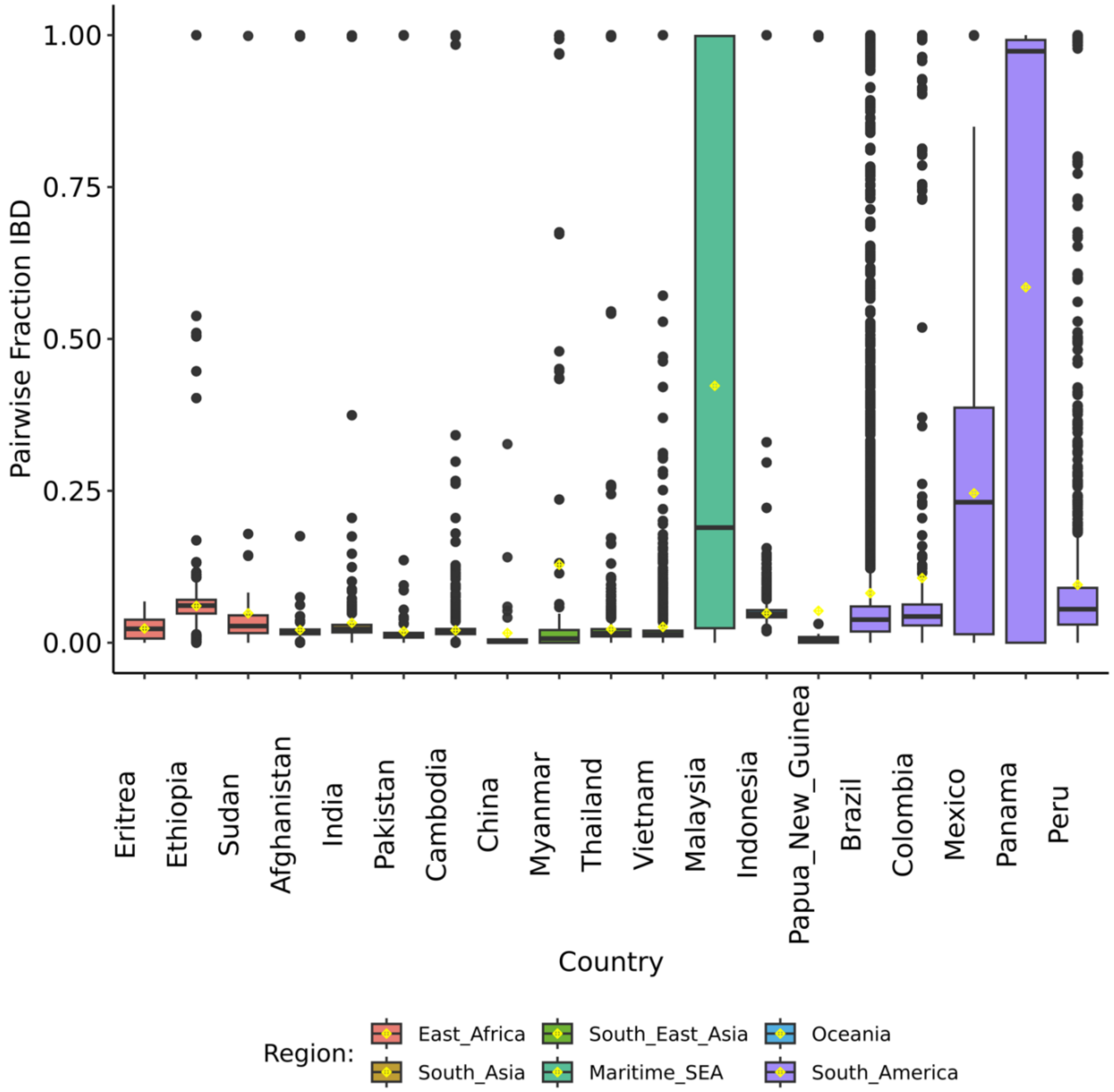

Figure S8. Genome-wide distribution of pairwise identity-by-descent (IBD) fractions at a country-level on chromosome 10 (positions 200,000 to 500,000) in *P. vivax* isolates.

Only populations with ≥10 monoclonal isolates are displayed. Boxes, coloured by subregional grouping, contain lines representing the median, lower quartile, and upper quartile. Whiskers extend to IBD values that are at a maximum of 1.5x the interquartile range. Outliers are represented as single points.

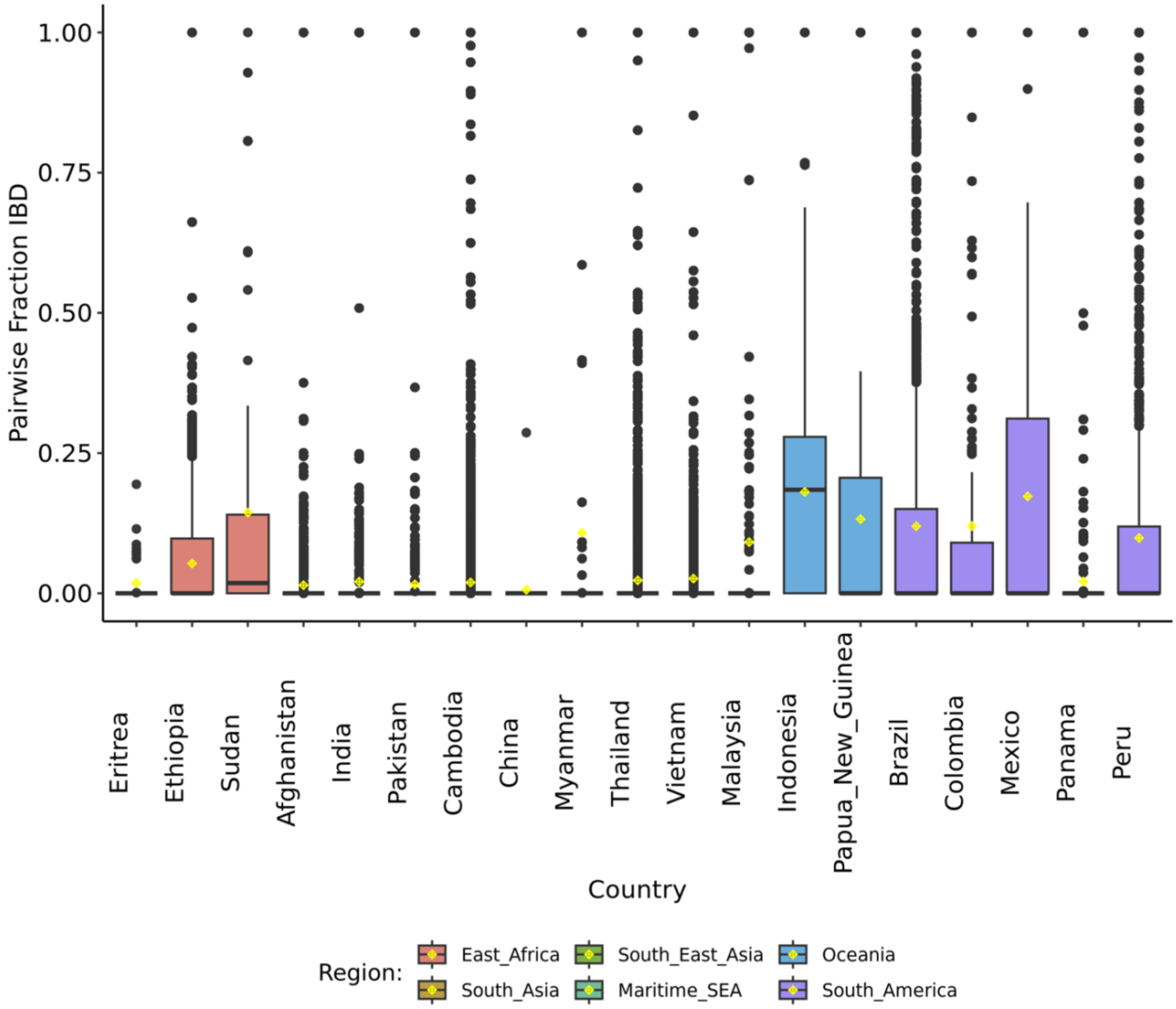

Figure S9. Genome-wide distribution of identity-by-descent (IBD) fractions calculated across sliding windows of 10 kb at a country-level on chromosome 10 (positions 200,000-500,000) in *P. vivax* isolates. Boundaries of regions of interest are denoted by vertical grey dashed lines.

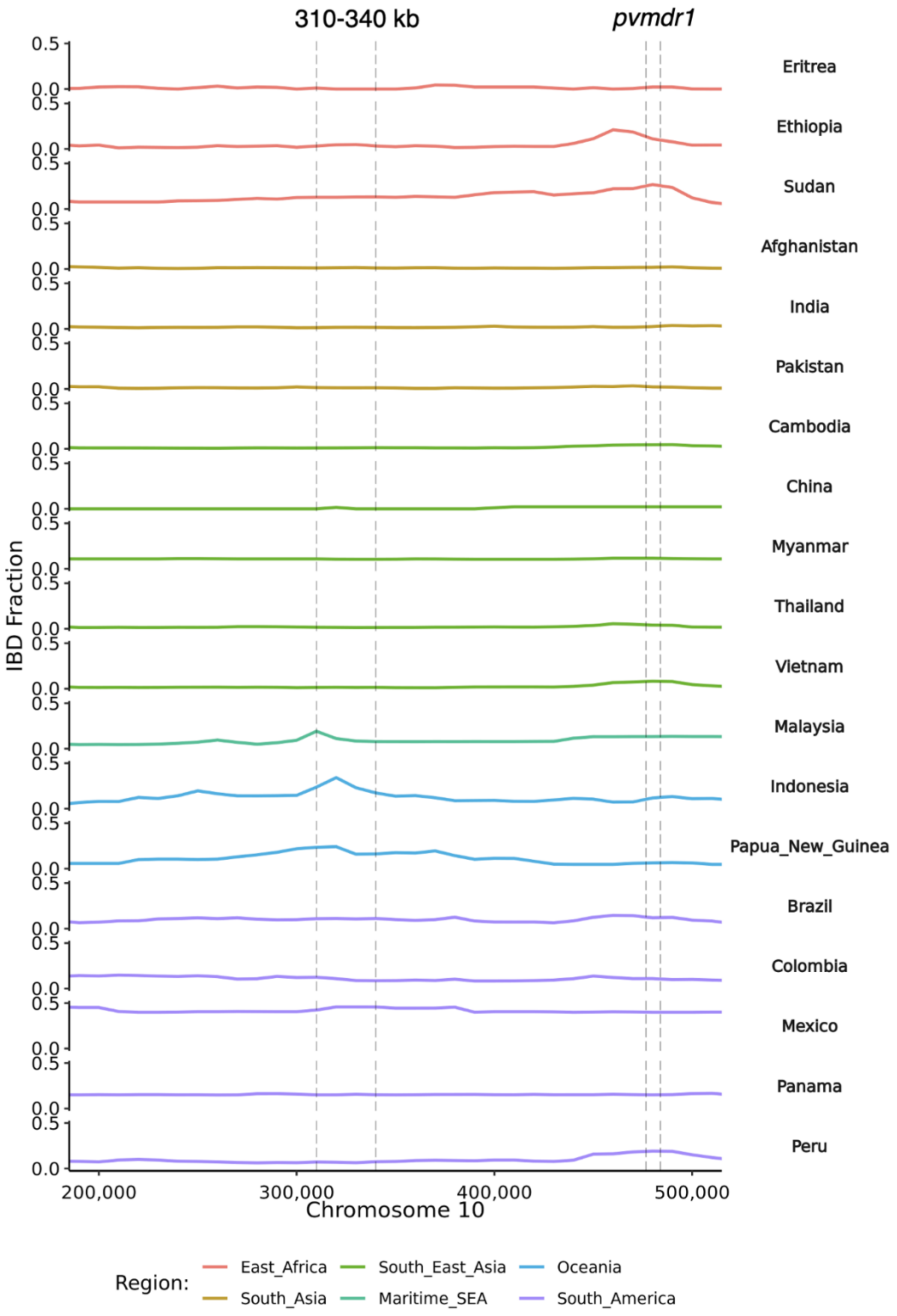

Figure S10. Genome-wide distribution of identity-by-descent (IBD) fractions calculated across sliding windows of 10 kb at a country-level on chromosome 10 in *P. vivax* isolates. Boundaries of regions of interest (240-340kb) are denoted by vertical grey dashed lines, and genes within this region are represented as a locus zoom.

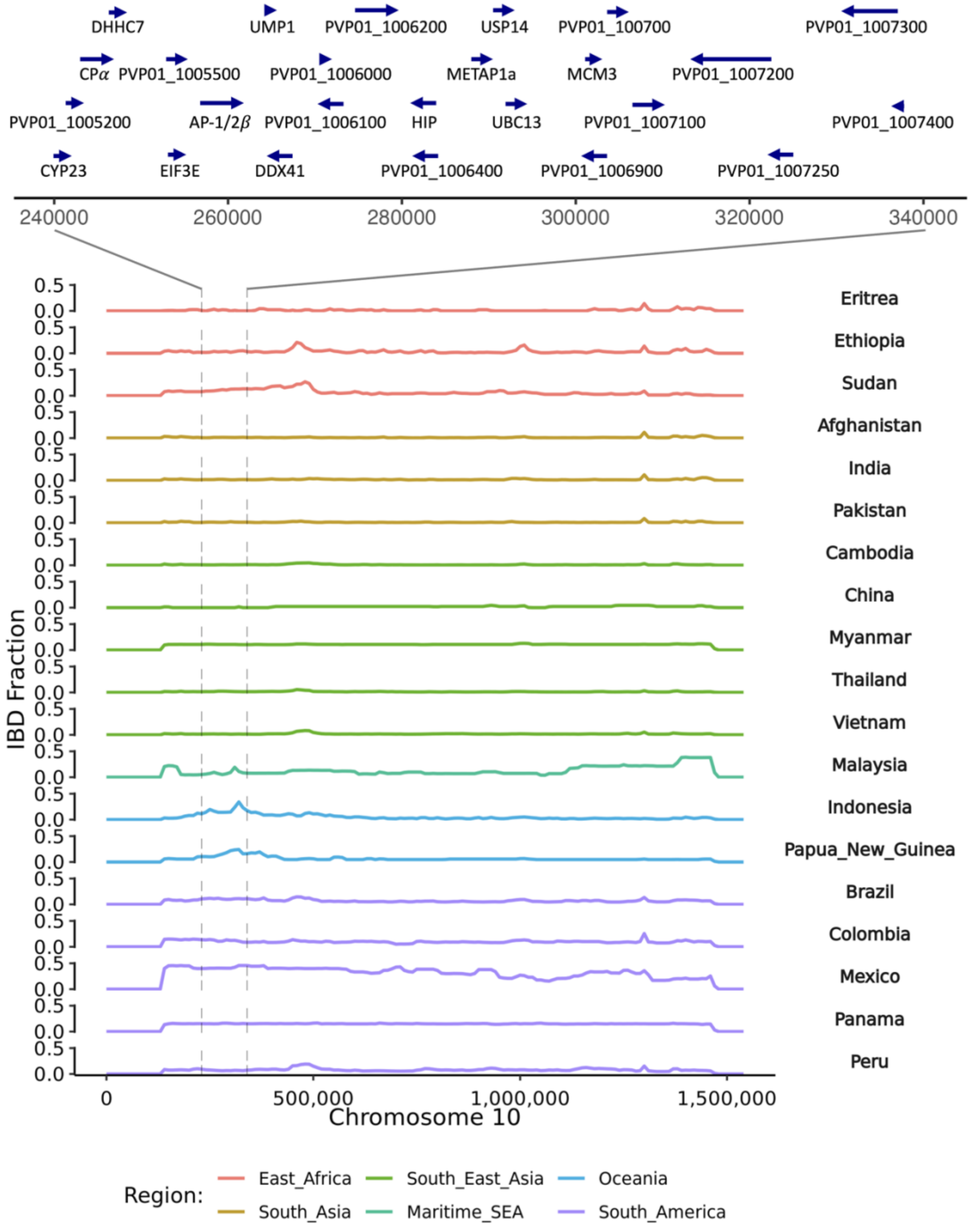

Figure S11. Protein structure predictions of PvMRP1 (PVP01_0203000) and PfMRP1 (PF3D7_0112200) amino acid mutations. Amino acid positions altered by SNPs are highlighted in blue for *P. vivax*, and red for *P. falciparum*. Aligned protein structures of PvMRP1 (with mutations present in Indonesian isolates) and PfMRP1 (with mutations known to be resistance-conferring) are shown in panels **a**-**d**, and where they lie in the: (**a**) ABC Transmembrane Domain 1, (**b**) AAA+ ATPase Domain 1, (**c**) ABC Transmembrane Domain 2, (**d**) and AAA+ ATPase Domain 2. A PvMRP1 and PfMRP1 sequence alignment is shown in (**e**), with residues highlighted in grey symbolising those with 100% sequence identity.

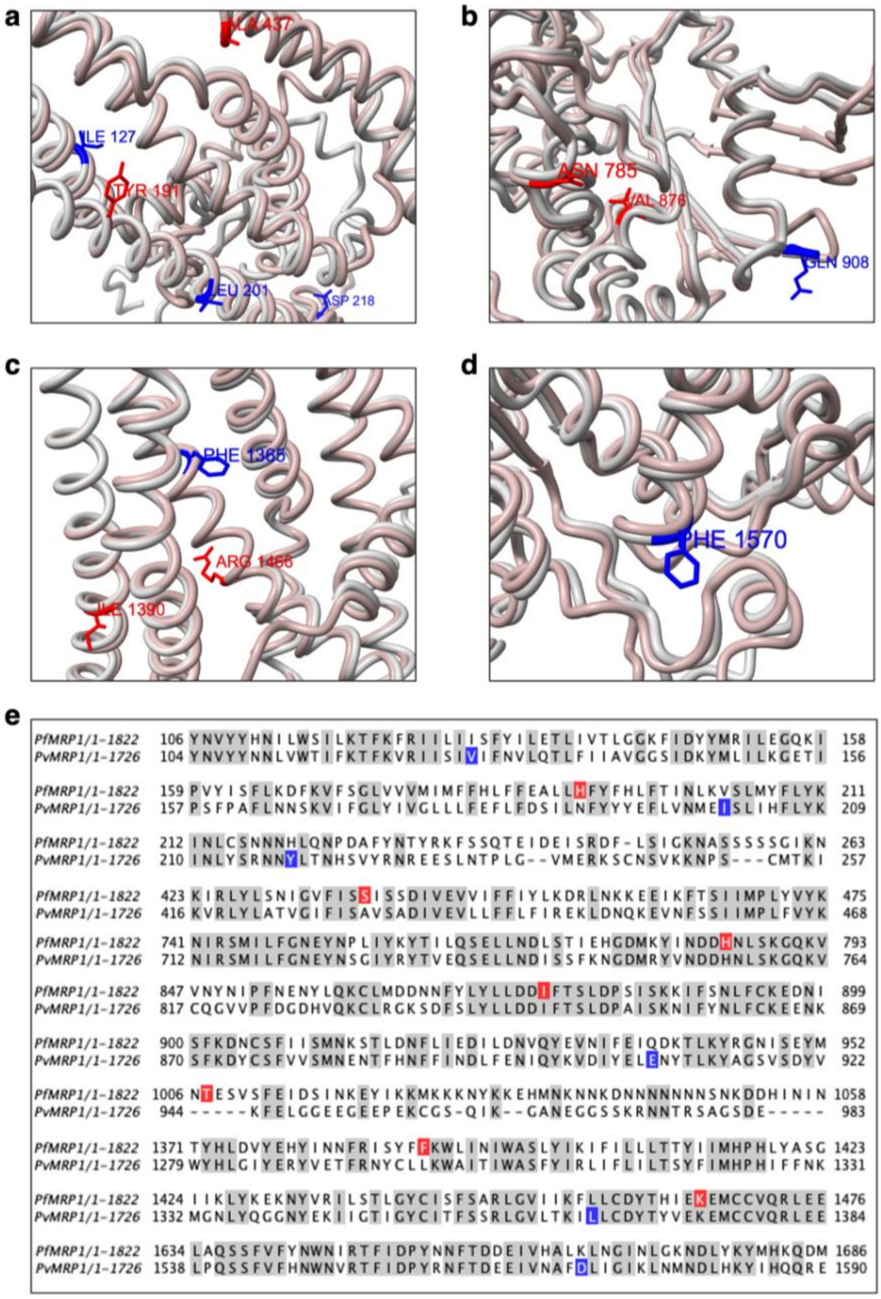

Figure S12. Transmembrane structure prediction of PvMRP1, showing transmembrane helices in navy numbered I-XII, and AAA+ ATPase domains in orange numbered I-II. Numbers within the transmembrane helices indicate the residue number that starts and ends the transmembrane helix. Numbers within the AAA+ ATPase domains indicate the residue number that starts and ends the transporter domain.

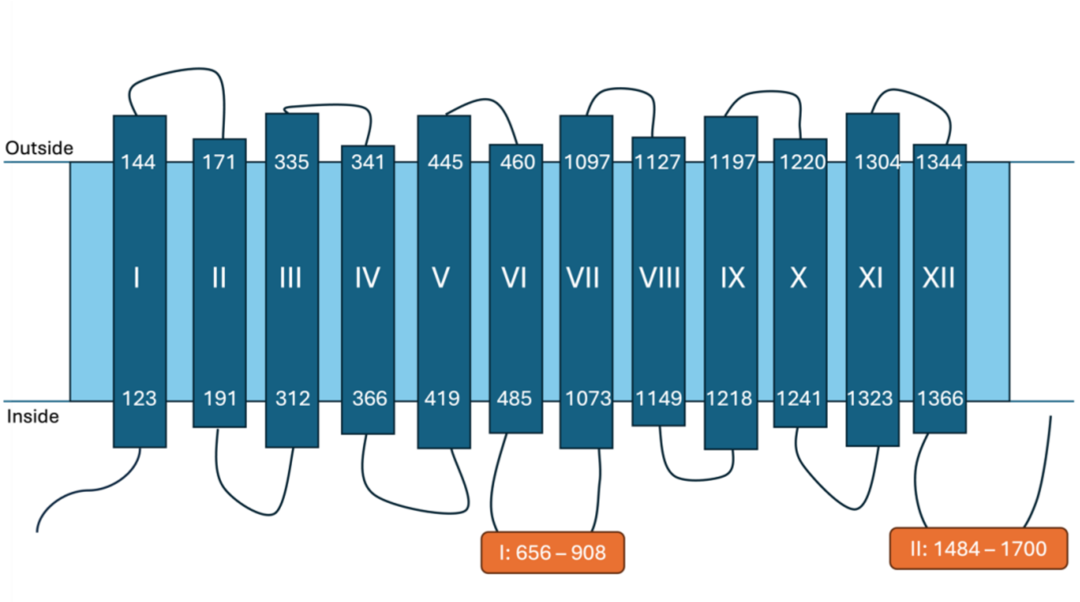

Figure S13. Manhattan plot of F_{ST} values between Indonesian Papua isolates from: (a) 2008-2009 vs. 2016-2017 and (b) Pre-2014 vs. Post-2014.

The red line symbolises F_{ST} values above the 99th percentile. SNPs within *pvmrp1* (L1365F and D1570F) are highlighted in red.

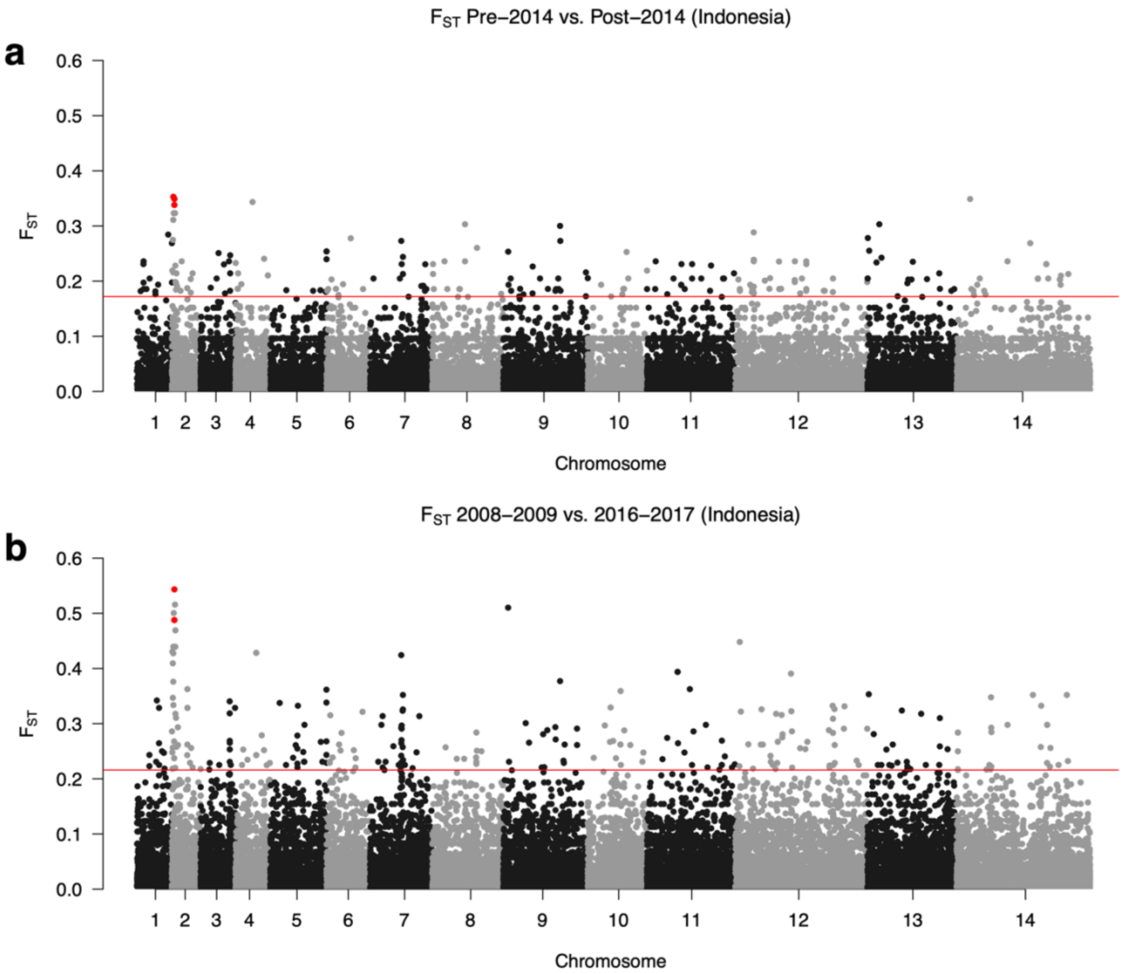

Figure S14. Median joining haplotype network constructed using upstream *pvmdr1* gene (Chromosome 10, Positions 243480-319423) sequences from 104 Indonesian Papua isolates.

Each node represents a haplotype, with each segment in the node representing isolates from a given regional grouping. Nodes are sized in proportion to the number of samples represented by that haplotype. The number of SNP differences between haplotypes is represented by the number of ticks between the nodes.

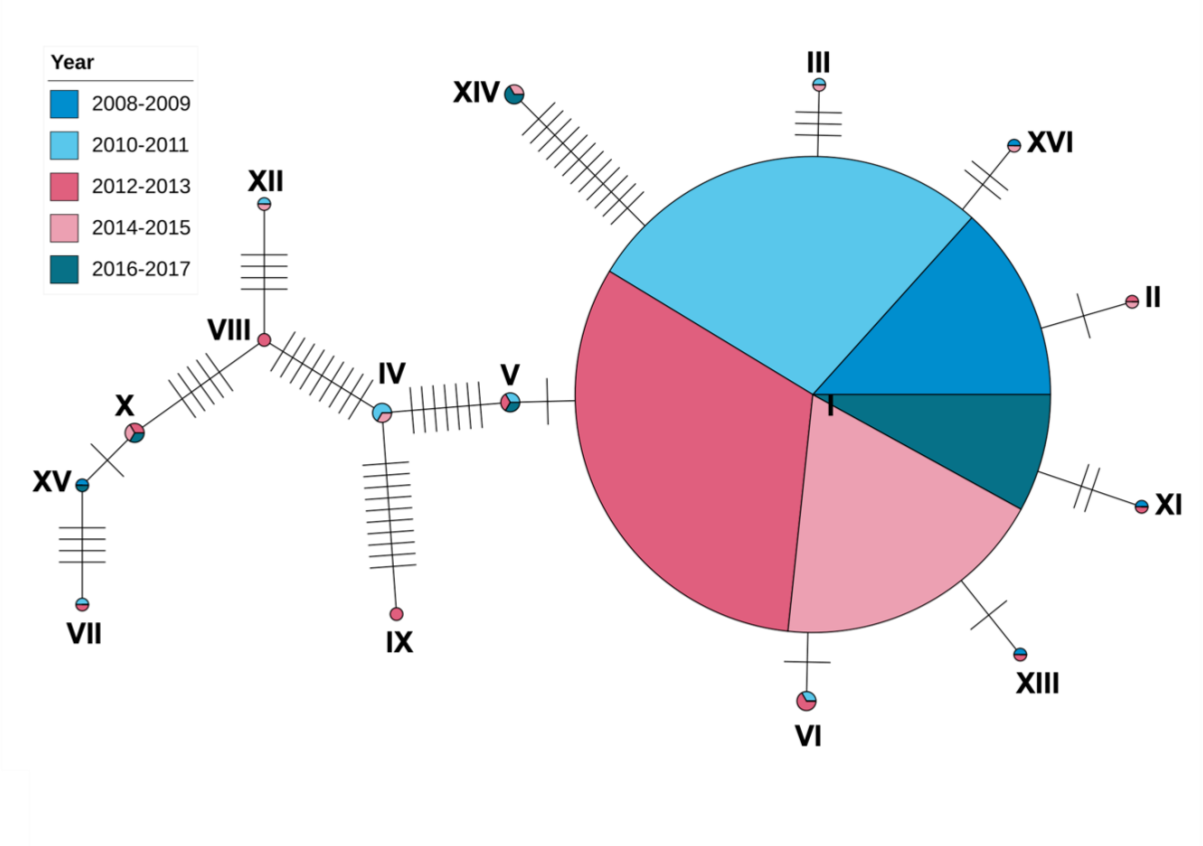

Supplement: Supplementary file 1 — Supplementary Information [file 41467_2024_54964_MOESM1_ESM.pdf]
