# Supplementary material for: Genomic analysis of global Plasmodium vivax populations reveals insights into the evolution of drug resistance
Source: Nat Commun. 2024 Dec 30;15:10771. doi: 10.1038/s41467-024-54964-x (PMC11685768; doi:10.1038/s41467-024-54964-x)
Supplement: Supplementary file 3 — Description of Additional Supplementary Files [file 41467_2024_54964_MOESM3_ESM.pdf]

Description of Additional Supplementary Files

File: Supplementary Data 1
Description: Samples, Provenance, and Accessions.

File: Supplementary Data 2
Description: Current and previous antimalarial use in *P. vivax* endemic countries with >10 samples available for analysis, and their corresponding chloroquine resistance (CQR) status. Countries now certified malaria-free have been excluded.

File: Supplementary Data 3
Description: All studies used to attribute chloroquine resistance status. "New" studies refer to those found in 2019-present date, post Price et al.'s 2014 meta-analysis and WWARN's Vivax Surveyor, using the same research methodology. Table was taken from WWARN's Vivax Surveyor and adapted to add new studies.

File: Supplementary Data 4
Description: Number (N) of Highly Differentiating SNPs with an FST value ≥ 0.8, ≥ 0.95, ≥ 0.99, and 1. SEA = South East Asia.

File: Supplementary Data 5
Description: Median Genome-wide identity by Descent (IBD) Fractions in all regions and countries with ≥10 monoclonal isolates ($F_{WS} \geq 0.95$). SEA= South East Asia.

File: Supplementary Data 6
Description: Inter-Regional Median Genome-Wide Identity by Descent (IBD) Fractions in Monoclonal Isolates ($F_{WS} \geq 0.95$). SEA = South East Asia.

File: Supplementary Data 7
Description: Top 1% of Regions with Highest Proportion of Pairwise Identity by Descent (IBD).

File: Supplementary Data 8
Description: Intra-Population Pairwise IBD at the 200-500 kb Region on Chromosome 10. SEA = South East Asia.

File: Supplementary Data 9
Description: Top 1% integrated Haplotype Homozygosity Scores (iHS, Within-Population Statistic) to Detect Selection in Countries with N ≥ 10. Loci in critical regions are defined here as SNPs in genes with a *p* value of $P < 1 \times 10^{-4}$ (two-sided test).

File: Supplementary Data 10
Description: Top 1% *Rsb* Scores (Between-Population Statistic) Between Countries with N ≥ 10 Each. Loci in critical regions are defined here as SNPs in genes with a *p* value of $P < 1 \times 10^{-5}$ (two-sided test).

File: Supplementary Data 11
Description: Top 1% *XP-EHH* Scores (Between-Population Statistic) Between Countries with N ≥ 10 Each. Loci in critical regions are defined here as SNPs in genes with a *p* value of $P < 1 \times 10^{-5}$ (two-sided test).

File: Supplementary Data 12
Description: Candidate regions of interest based on Top 1% *iHS*, *Rsb,* XP-EHH*, and IBD* Scores when comparing Indonesian Papua isolates (high-grade chloroquine resistance) with regions that are chloroquine sensitive.

File: Supplementary Data 13
Description: Non-Synonymous Mutations in Putative Drug Resistance-Associated Loci

File: Supplementary Data 14
Description: Haplotypes in *pvdhfr, pvdhps, pvmrp1*, and *pvmdr1* in monoclonal isolates.

File: Supplementary Data 15
Description: Nucleotide diversity (*pi*) calculated in 100 bp windows and haplotype diversity (*h*) of *pvmdr1*

File: Supplementary Data 16
Description: Top 1% of FST values (Population Differentiation) between Indonesian Papua isolates between 2008-2008 vs. 2016-2017 and Pre-2014 vs. Post-2014.

File: Supplementary Data 17
Description: Top 1% integrated Haplotype Homozygosity Scores (*iHS*, Within-Population Statistic) to Detect Selection in Indonesian Papua isolates categorised by year groupings (2008-09, 2010-11, 2012-13, 2014-2015, 2016-17) and Pre vs. or Post 2014 Groupings. Loci in critical regions are defined here as SNPs in genes with a *p* value of $P < 1 \times 10^{-4}$ (two-sided test).

File: Supplementary Data 18
Description: Top 1% *Rsb* Scores (Between-Population Statistic) to Detect Selection Between Indonesian Papua isolates categorised by year groupings (2008-09, 2010-11, 2012-13, 2014-15, 2016-17). Loci in critical regions are defined here as SNPs in genes with a *p* value of $P < 1 \times 10^{-5}$ (two-sided test).

File: Supplementary Data 19
Description: Top 1% *XP-EHH* Scores (Between-Population Statistic) to Detect Selection Between Indonesian Papua isolates categorised by year groupings (2008-09, 2010-11, 2012-13, 2014-15, 2016-17). Loci in critical regions are defined here as SNPs in genes with a *p* value of $P < 1 \times 10^{-5}$ (two-sided test).
