# Supplementary material for: Genomic analysis of global Plasmodium vivax populations reveals insights into the evolution of drug resistance
Source: Nat Commun. 2024 Dec 30;15:10771. doi: 10.1038/s41467-024-54964-x (PMC11685768; doi:10.1038/s41467-024-54964-x)
Supplement: Supplementary file 23 — Reporting Summary [file 41467_2024_54964_MOESM23_ESM.pdf]

nature portfolio

Corresponding author(s): Taane Clark and Susana Campino

Last updated by author(s): 10/10/2024

Reporting Summary

Nature Portfolio wishes to improve the reproducibility of the work that we publish. This form provides structure for consistency and transparency in reporting. For further information on Nature Portfolio policies, see our Editorial Policies and the Editorial Policy Checklist.

Statistics

For all statistical analyses, confirm that the following items are present in the figure legend, table legend, main text, or Methods section.

n/a	Confirmed	
☐	☒	The exact sample size (*n*) for each experimental group/condition, given as a discrete number and unit of measurement
☒	☐	A statement on whether measurements were taken from distinct samples or whether the same sample was measured repeatedly
☐	☒	The statistical test(s) used AND whether they are one- or two-sided<br>*Only common tests should be described solely by name; describe more complex techniques in the Methods section.*
☒	☐	A description of all covariates tested
☒	☐	A description of any assumptions or corrections, such as tests of normality and adjustment for multiple comparisons
☐	☒	A full description of the statistical parameters including central tendency (e.g. means) or other basic estimates (e.g. regression coefficient) AND variation (e.g. standard deviation) or associated estimates of uncertainty (e.g. confidence intervals)
☐	☒	For null hypothesis testing, the test statistic (e.g. *F*, *t*, *r*) with confidence intervals, effect sizes, degrees of freedom and *P* value noted<br>*Give P values as exact values whenever suitable.*
☒	☐	For Bayesian analysis, information on the choice of priors and Markov chain Monte Carlo settings
☒	☐	For hierarchical and complex designs, identification of the appropriate level for tests and full reporting of outcomes
☒	☐	Estimates of effect sizes (e.g. Cohen's *d*, Pearson's *r*), indicating how they were calculated

Our web collection on statistics for biologists contains articles on many of the points above.

Software and code

Policy information about availability of computer code

Data collection	No software was used for data collection.
Data analysis	For bioinformatics analyses, we used trimmomatic (version 0.39), bwa-mem (version 0.7.17), samtools (version 1.9), and GATK (version 4.4.0.0). For population genetics analyses, we used snpEFF (version 5.1), PLINK (version 1.9), ape (version 5.7), bcftools (version 1.17), R (version 4.2.3), vcftools (version 0.1.17), moimix (version 0.0.2.9001), ADMIXTURE (version 1.3.0), hmmIBD (version 2.04), rehh (version 3.2.2), pegas (version 0.11). For protein structural analysis we used Clustal Omega (https://www.ebi.ac.uk/jdispatcher/msa/clustalo, accessed August 2024) , AlphaFold3 (https://alphafoldserver.com, accessed August 2024) DeepTMHMM (https://dtu.biolib.com/DeepTMHMM, accessed August 2024), InterPro (https://www.ebi.ac.uk/interpro/, accessed August 2024), JalView (version 2.11.3.3) and UCSF ChimeraX (version 1.17.3).

For manuscripts utilizing custom algorithms or software that are central to the research but not yet described in published literature, software must be made available to editors and reviewers. We strongly encourage code deposition in a community repository (e.g. GitHub). See the Nature Portfolio guidelines for submitting code & software for further information.

Data

Policy information about availability of data

All manuscripts must include a data availability statement. This statement should provide the following information, where applicable:

- Accession codes, unique identifiers, or web links for publicly available datasets
- A description of any restrictions on data availability
- For clinical datasets or third party data, please ensure that the statement adheres to our policy

The data used in this study are available at the European Nucleotide Archive (https://www.ebi.ac.uk/ena). For samples from imported P. vivax cases diagnosed at the UKHSA Malaria Reference Laboratory, data are under accession codes PRJEB44419 and PRJEB56411. For samples from the MalariaGEN P. vivax Genome Variation Project54, data are under accession codes PRJEB2136, PRJEB2140, PRJEB4409, PRJEB4410, PRJEB10888, PRJNA65119, PRJNA67065, PRJNA67237, PRJNA67239, PRJNA175266, PRJNA240366-240531, PRJNA271480, PRJNA284437, PRJNA295233, PRJNA420510, PRJNA432819, PRJNA603279, PRJNA643698, PRJNA655141. The raw data for 175 Indonesian Papua isolates from the MalariaGEN P. vivax Genome Variation Project are available as an unfiltered VCF at ftp://ngs.sanger.ac.uk/production/malaria/Resource/30. Accession codes and sample provenance are detailed in Table S1.

Research involving human participants, their data, or biological material

Policy information about studies with human participants or human data. See also policy information about sex, gender (identity/presentation), and sexual orientation and race, ethnicity and racism.

Reporting on sex and gender	No human participants or human data were used or involved in this study.
Reporting on race, ethnicity, or other socially relevant groupings	No human participants or human data were used or involved in this study.
Population characteristics	No human participants or human data wee used or involved in this study.
Recruitment	All samples are publicly available, however, ethical approval was obtained for using sequence data from adult travelers entering the UK with a positive vivax malaria diagnosis (UK Health Security Agency Malaria Reference Laboratory at the London School of Hygiene and Tropical Medicine).
Ethics oversight	LSHTM Research Ethics Committee (REF: 14710) and the UK National Research Ethics Service (REF: 18/LO/0738).

Note that full information on the approval of the study protocol must also be provided in the manuscript.

Field-specific reporting

Please select the one below that is the best fit for your research. If you are not sure, read the appropriate sections before making your selection.

[x] Life sciences [ ] Behavioural & social sciences [ ] Ecological, evolutionary & environmental sciences

For a reference copy of the document with all sections, see nature.com/documents/nr-reporting-summary-flat.pdf

Life sciences study design

All studies must disclose on these points even when the disclosure is negative.

Sample size	No sample size calculations were made. We maximised numbers of samples by using a convenience sampling framework.
Data exclusions	Poor quality sequence data and samples with poor genomic coverage or missingness (>40%) excluded.
Replication	Not applicable, as all samples were used for primary analyses.
Randomization	Not applicable, as this is a cohort analysis.
Blinding	Not applicable, as this is a cohort analysis.

Reporting for specific materials, systems and methods

We require information from authors about some types of materials, experimental systems and methods used in many studies. Here, indicate whether each material, system or method listed is relevant to your study. If you are not sure if a list item applies to your research, read the appropriate section before selecting a response.

Materials & experimental systems

n/a	Involved in the study
☒	☐ Antibodies
☒	☐ Eukaryotic cell lines
☒	☐ Palaeontology and archaeology
☒	☐ Animals and other organisms
☒	☐ Clinical data
☒	☐ Dual use research of concern
☒	☐ Plants

Methods

n/a	Involved in the study
☒	☐ ChIP-seq
☒	☐ Flow cytometry
☒	☐ MRI-based neuroimaging

Plants

Seed stocks	*Report on the source of all seed stocks or other plant material used. If applicable, state the seed stock centre and catalogue number. If plant specimens were collected from the field, describe the collection location, date and sampling procedures.*
Novel plant genotypes	*Describe the methods by which all novel plant genotypes were produced. This includes those generated by transgenic approaches, gene editing, chemical/radiation-based mutagenesis and hybridization. For transgenic lines, describe the transformation method, the number of independent lines analyzed and the generation upon which experiments were performed. For gene-edited lines, describe the editor used, the endogenous sequence targeted for editing, the targeting guide RNA sequence (if applicable) and how the editor was applied.*
Authentication	*Describe any authentication procedures for each seed stock used or novel genotype generated. Describe any experiments used to assess the effect of a mutation and, where applicable, how potential secondary effects (e.g. second site T-DNA insertions, mosiacism, off-target gene editing) were examined.*
